# Markers of Oxidative Stress and Antioxidant Defense in Romanian Patients with Type 2 Diabetes Mellitus and Obesity

**DOI:** 10.3390/molecules22050714

**Published:** 2017-05-01

**Authors:** Ariana Picu, Laura Petcu, Simona Ştefan, Manuela Mitu, Daniela Lixandru, Constantin Ionescu-Tîrgovişte, Grațiela Grădișteanu Pîrcălăbioru, Felicia Ciulu-Costinescu, Maria-Viorica Bubulica, Mariana Carmen Chifiriuc

**Affiliations:** 1 NIDNMD “Prof. N.C. Paulescu”, 2th district, Bucharest 020042, Romania; arianapicu@gmail.com (A.P.); simona_ds2002@yahoo.com (S.S.); mani_mitu2002@yahoo.co.uk (M.M.); cit@paulescu.ro (C.I.-T.); 2 Faculty of Biology, University of Bucharest, 5th district, Bucharest 050095, Romania; gratiela87@gmail.com (G.G.P.); carmen.chifiriuc@gmail.com (M.C.C.); 3 Department of Biochemstry, University of Medicine and Pharmacy “Carol Davila”, 5th district, Bucharest 050474, Romania; danielalixandru@gmail.com; 4 Research Institute of the University of Bucharest, ICUB, 5th district, Bucharest 050107, Romania; felicia.costinescu@yahoo.com; 5 Faculty of Pharmacy, University of Medicine and Pharmacy of Craiova, Petru Rareș Str., Craiova 200638, Romania; mariaviorica.bubulica@gmail.com

**Keywords:** oxidative stress, type 2 diabetes mellitus, obesity, reactive oxygen species, inflammation

## Abstract

Type 2 diabetes mellitus (T2DM) is strongly associated with obesity. The adipose tissue secretes bioactive adipokines leading to low grade inflammation, amplified by oxidative stress, which promotes the formation of advanced glycation end products and eventually leads to dyslipidemia and vascular complications. The aim of this study was to correlate anthropometric, biochemical and oxidative stress parameters in newly diagnosed (ND) T2DM patients and to investigate the role of oxidative stress in T2DM associated with obesity. A group of 115 ND- T2DM patients was compared to a group of 32 healthy subjects in terms of clinical, anthropometric, biochemical and oxidative stress parameters. ND-T2DM patients had significantly lower adiponectin, glutathione (GSH) and gluthatione peroxidase (GPx) and elevated insulin, proinsulin, HOMA-IR index, proinsulin/insulin (P/I) and proinsulin/adiponectin (P/A) ratio, fructosamine, and total oxidant status (TOS). The total body fat mass was positively correlated with total oxidant status (TOS). Positive correlations were found between TOS and glycated hemoglobin (HbA1c), and between TOS and glycaemia. Negative correlations were identified between: GPx and glycaemia, GPx and HbA1c, and also between GSH and fructosamine. The total antioxidant status was negatively correlated with the respiratory burst. The identified correlations suggest the existence of a complex interplay between diabetes, obesity and oxidative stress.

## 1. Introduction

Currently, diabetes can be considered an epidemic disease, with a high, biological, socio-economic and political impact. Its increasing prevalence worldwide, correlated with that of overweight and obesity, is most commonly associated in adults with type 2 diabetes mellitus (T2DM), which is often called nowadays “diabesity”. The pathogenic mechanisms of diabetes and its complications are incompletely elucidated, the disease being characterized by a lack of curative therapies [1]. 

The World Health Organization (WHO) report presented in Geneva in 2016 claims that the number of people with diabetes is now four times higher than in 1980, numbering 422 million diabetics worldwide [2]. Eighty percent of patients with T2DM are obese. Eurostat data showed a prevalence of obesity in the adult population of the European Union of 17.2%, with a prevalence of overweight of 35.9% (http://epp.eurostat.ec.europa.eu/statistics_explained/index.php/Overweight_ and_obesity_-_BMI_statistics). In Romania, the epidemiological Prevalence of Diabetes, Prediabetes, Overweight, Obesity, Dyslipidemia and Chronic Kidney Disease (PREDATORR) study, performed in 2013, revealed a prevalence of 11.6% corresponding to 1.752 million diabetic patients, out of which 20.69% are newly diagnosed [3]. According to the same study only 31.7% of the Romanian population has a normal weight, the remaining of 34% being overweight and 31.4% obese. 

The effect of obesity on the pathology of T2DM depends on the one hand on the central or peripheral distribution of fat tissue and on the other hand on the "aggressive" adipocytes phenotype, with intense secretory function, producing more than 100 various signal molecules, the adipose tissue being thus considered as "the third brain" and "a tissue with high intelligence quotient". The abdominal or central obesity which includes the visceral fat maintains and worsens the metabolic dysfunction in T2DM. Insulin resistance is a hallmark of T2DM. Because plasma insulin levels increase with increasing body weight, almost all overweight/obese patients are characterized as insulin resistant. In subjects without diabetes, insulin resistance is associated with a clustering of cardiovascular risk factors and a high incidence of cardiovascular disease [4,5] approximating the cardiovascular risk of patients with T2M [6,7]. Insulin resistance, therefore, has been proposed to be the common link between T2M and cardiovascular disease [8].

The increased endogenous glucose output (EGO) occurring in T2DM leads to an imbalance in the production of free radicals and antioxidants. The increased oxidative stress in T2DM is accompanied by the formation of advanced glycation end products (AGEs) leading to dyslipidemia, inflammatory, vascular, and thrombotic complications. The “adipocyte dysfunction" in obesity also involves the occurrence of oxidative stress and of prothrombotic risk [9]. Briefly, adipocyte dysfunction can be described as an energy imbalance (excessive intake of nutrients) with the occurrence of dyslipidemia, adipocytes hypertrophy and hyperplasia; infiltration of adipose tissue with monocyte-macrophages, increased secretion of pro-inflammatory cytokines and high levels of adipokines inducing overloading of the endoplasmic reticulum (ER), which leads to the following processes: ER dysfunction, accumulation of incorrectly folded proteins, adipocyte apoptosis amplifying the inflammatory cascade [10]. ER stress along with mitochondrial oxidative stress causes further increased production of reactive oxygen species, which leads to insulin resistance by decreasing the intracellular glucose transport [11]. The excess glucose determines an enhancement in the production of free radicals which inhibit the activity of GLUT4 (intracellular glucose transporter sensitive to insulin), amplify the oxidative stress (by increasing the oxidant status and the respiratory burst, decreasing the antioxidant capacity exerted by superoxide dismutase, glutathione peroxidase, glutathione or both mechanisms) and enhance insulin resistance [12,13]. The intensification of the oxidative stress, due to both the chronic hyperglycemia and persistent circulating triglyceride-rich lipoproteins [14] is closely linked to alterations in monocyte and macrophage function [15].Oxidative stress and inflammation, especially associated with obesity, coexist and reinforce each other eventually leading to insulin resistance, beta-pancreatic cells dysfunction and vascular complications in T2DM [16].The aim of this study was to investigate the role of oxidative stress in the pathogenesis of T2DM associated with obesity.

## 2. Results 

Increased values of certain anthropometric parameters (weight, waist circumference, body mass index, body fat percentage and the visceral fat level) in diabetic patients compared to control group are due to the well-known association of T2DM with obesity. Thus, the perivisceral obesity leads to a decrease in peripheral sensitivity to insulin, especially in patients with reduced glycemic control. The clinical and anthropometrical characteristics of the healthy subjects and patients with T2DM divided by gender are shown in Table 1.

The routine biochemistry measurements for both groups—healthy subjects and T2DM—are presented in Table 2. Regarding serum fasting glucose and glycated hemoglobin a significant increase in concentration of these parameters in patients with T2DM compared with controls (*p* < 0.001) was observed. Referring to the lipid profile of studied patients we found statistically significant elevated values for serum triglycerides, Triglyceride/HDLc ratio (atherogenic index, A.I.) (*p* < 0.001) and Total Chol./HDLc ratio (*p* < 0.05) in diabetic subjects compared to the control group, while serum HDLc was significantly decreased compared to healthy subjects (*p* < 0.05).

We also found statistically significant *p*-values for the triglycerides to HDLc ratio when comparing females from the CG versus females from the ND-T2DM group (*p* < 0.001) and regarding the comparison between males from both studied groups the p-values were also found to be statistically significant (*p* < 0.05). The mean values for the triglycerides to HDLc ratio were elevated for both control group males and diabetic males comparing to females. A possible explanation may be that although their body fat mass values were lower, values for weight, waist circumference and visceral fat level in the male category were higher than in females. This suggests again the role of obesity as a main risk factor and especially of the visceral adipose tissue, in the etiopathology of T2DM. The differences for total serum cholesterol and LDLc between the studied groups were not statistically significant, these parameters having elevated values for both groups.

Values of fructosamine and glycated hemoglobin were higher in diabetics versus healthy subjects with statistically significant differences between these two groups, which shows an increased degree of glycation for both hemoglobin and other proteins, mainly albumin, accelerating production of AGEs increasing the oxidative stress that leads to activation of inflammatory signaling molecules with pathogenic effects in diabetes and its complications [17]. Fructosamine determines total serum protein fraction which has undergone glycation and because albumin is the most abundant protein in the blood, levels of fructosamine reflects the degree of glycation of albumin. Because the half-life of albumin is about 20 days, serum fructosamine concentrations reflect recent changes in blood glucose for the last 1–2 weeks. From the biological point of view, fructosamines are recognized by the fructosamine-3-kinase which triggers the degradation of AGEs.

Exposure to AGEs activates endothelial cell adhesion molecule synthesis, pro-coagulant factors and lowers the level of cellular glutathione. Receptors for AGEs (RAGE) are involved in the migration and activation of monocytes, providing macrophages the ability to bind erythrocytes modified by glycation [18,19,20].

The values of parameters for assessing pancreatic beta cells function, adipocytes function and parameters for the evaluation of the oxidant/antioxidant status for the healthy and diabetic subjects, as well as comparisons between the two groups are shown in Table 3.

Although leptin to adiponectin ratio (L/A) is a useful measure of insulin resistance both in diabetic and non-diabetic subjects [21,22,23,24,25] to predict the presence of the metabolic syndrome and cardiovascular risk [26,27], we also find the proinsulin to adiponectin ratio (P/A), the first component indicating β-cell and the later adipocyte cell dysfunction, a useful early indicator of T2DM associated with obesity. This ratio increases significantly in T2DM because proinsulin increases progressively, while adiponectin gradually decreases [28]. Mainly seen as an indicator for impaired β-cell function, proinsulin can be detected at low concentrations in the blood of healthy persons but is found at higher concentrations in the blood of insulin-resistant subjects [29] and patients with type 2 diabetes [30,31,32]. Adiponectin is a protein expressed in adipose tissue and can be detected at concentrations of approximately 7–12 mg/L in human blood. In contrast to proinsulin, the concentration of adiponectin is suppressed in patients with T2DM and especially in patients with additional cardiovascular complications [33]. Based on these results, both substances seem to be indicators of insulin resistance and high cardiovascular risk in patients with type 2 diabetes.

The values of parameters for evaluation of pancreatic beta cells function showed a statistically significant increase in insulin, proinsulin, HOMA-IR index, proinsulin/insulin (P/I) and proinsulin/adiponectin (P/A) ratio (*p* < 0.001) in diabetic patients compared to the control group. This increase confirms the theory of insulin resistance associated with hyperinsulinemia that promotes an elevation in free radicals generation by NADPH-dependent mechanisms [34].

The parameters of the secretory function of the adipose tissue showed an increase, although not statistically significant, of leptin levels and a statistically significant decrease (*p* < 0.05) of adiponectin values in diabetics compared to the control group.

**Figure 1:**
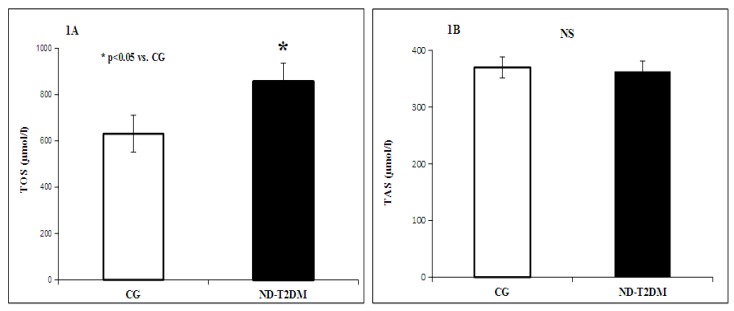
Total oxidant status (TOS) (**A**) total antioxidant status (TAS) (**B**) in patients with ND-T2DM versus control group (CG).

Regarding the values of the parameters that characterize the oxidant/antioxidant balance, they were higher in diabetic patients compared to healthy subjects, being statistically significant, resulting in a *p* < 0.05 for the total oxidant status (TOS). It has also been observed a decrease in the levels of superoxide dismutase (SOD) and slightly low values (not statistically significant) for the total antioxidant status (TAS) in diabetics compared with healthy subjects, probably indicating a tendency of depletion of the body’s antioxidants in an attempt to counterbalance the intensive oxidative stress (Figure 1A,B).

**Figure 2:**
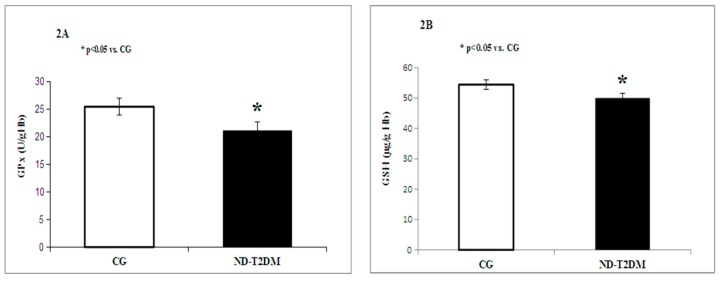
Circulating levels of glutathione peroxidase (GP_x_) (**A**) and glutathione (GSH) (**B**) in patients with ND-T2DM versus control group (CG).

Glutathione peroxidase (GPx) and glutathione (GSH) (*p* < 0.05) were significantly decreased in diabetics versus healthy subjects (Figure 2A,B).

**Figure 3:**
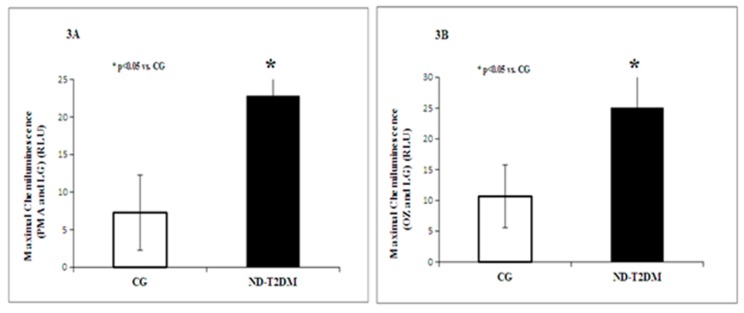
Respiratory burst (PMA and LG) (**A**) and (OZ and LG) (**B**) in patients with ND-T2DM versus control group (CG).

The intensity of respiratory burst (RB) was increased after stimulation with phorbol-12myristate-13acetate (PMA) and with opsonized zymosan (OZ) of PBMCs isolated from diabetic patients versus healthy subjects (*p* < 0.05) (Figure 3A,B).

## 3. Discussion

The “perpetuum” of diabetes begins with an initial defect of insulin action followed by a decrease of beta cell function. When fasting plasma glucose (FPG) increases above diabetic level it is considered that the level of beta cells secretion is decreased with 80% and insulin resistance is at a maximum level [35]. The balance between these pathogenic mechanism and other factors such as the excess and distribution of fat tissue, its pro-inflammatory state or secreted adipokines is particular for each clinical situation and explains the metabolic diversity of type 2 diabetes (T2DM) [36,37].

Type 2 diabetes associated with obesity leads to the establishment of peripheral insulin resistance. Intensification of insulin resistance causes a compensatory stimulation of secretory function of pancreatic β cells trying to maintain glucose homeostasis. The persisting secretion leads to the depletion of functional pancreatic cells due to apoptosis of beta cells with progressive worsening of diabetes and to micro- and macrovascular complications. Obesity associated with T2DM enhances the pro-inflammatory status due to some specific features of the adipose tissue, like hypertrophy, hyperplasia, a peculiar fat distribution, predominantly abdominal (perivisceral), an increased secretion of adipokines and infiltration of macrophages. The fat cells change from physiologically “silent” to an “aggressive” phenotype.

One of the adipocytokines secreted by the adipose tissue is leptin, which has structural and functional similarities with classics pro-inflammatory cytokines thus leading to the proliferation of monocytes and an increased activity of NADPH oxidase consequently contributing to the release of reactive oxygen species (ROS), like superoxide anion and hydroperoxide radical.

The cellular constituents of the adipose tissue secrete a large number of bioactive adipokines, not only leptin and adiponectin, that have endocrine, paracrine and autocrine effects. Adipocytes and adipose tissue-derived macrophages are the source of the several circulating mediators of inflammation. Excess or unbalanced secretion of adipokines, a frequent occurrence in obesity, induces an inflammatory state also known as "low grade inflammation", which in turn amplifies the peripheral insulin resistance and has multiple effects on endothelial cells function. 

There are several hypotheses that attempted to explain the infiltration of adipose tissue with pro-inflammatory cells, the most widely accepted is the hypoxia hypothesis proposed in 2008 by Trayhurn and Wood [38]. An important decrease in weight can attenuate the pro-inflammatory response of adipocytes with "aggressive" adipocytes regressing to the status of "restless" adipocytes and the restless to their natural state of "quiet" adipocytes [39].

Many studies have focused on monocytes/macrophages function which by their respiratory burst produce free radicals that can initiate the atherogenic processes, with the formation of "foam cells" in the vascular endothelium leading to vascular complications of T2DM [40,41]. In T2M, release of free radicals during the respiratory burst of peripheral blood mononuclear cells (PBMC) is proportional to the level of blood glucose [42,43].

Oxidation processes constantly occur in aerobic biological systems, and even in case of a normal redox status, the antioxidant systems cannot completely eliminate reactive oxidizing species (oxidants) [44].

The mechanisms involved in the generation of oxidative stress at the cellular level involves the autoxidation of glucose, modification of the polyol pathway by increasing sorbitol, processes of lipid peroxidation, glycation with the accumulation of AGEs which stimulate the synthesis of interleukin-1 (IL-1) and fibroblast proliferation with subsequent endothelial damage, modification of the hexosamines pathway that induces the formation of fructose-6-phosphate, a substrate for protein glycosylation with the production of proteoglycans and the last but not least the intensification of respiratory burst processes with the depletion of NADPH oxidase [45].

NADPH oxidase is not only the source of reducing equivalents for respiratory burst reactions but is also involved in the detoxification pathway of glutathione. This compound is recovered from NADP by glucose-6-phosphate dehydrogenase (G6PD) in hexozo-monophosphate path.

In our experiments, the activation of NADPH oxidase 2(Nox2) was measured in mononuclear cells isolated from peripheral blood by using opsonised zymosan (a mimetic of a pathogen from the yeast *Saccharomyces cerevisiae* and a strong NADPH oxidase activator). Although opsonised zymosan is not a stimulus for monocyte activation in vivo, it is possible that in situ, these adjustment and control pathways to be the same.

At the time of activation of monocytes with opsonised zymosan the NADPH oxidase cytosolic components translocate to join the membrane components (gp91phox and p22phox). Determination of phagocyte oxidative burst intensity can be achieved in vitro by chemiluminescence method using luminol/lucigenin and stimulation with opsonized zymosan or phorbol 12-myristate 13-acetate (PMA). PMA is a structural analogue of the diacylglycerol (DAG), which directly phosphorylates protein kinase C (PKC) activating it, leading ultimately to the activation of NADPH oxidases and producing a large amount of reactive oxygen species (ROS). NADPH oxidase activation after PMA stimulation occurs on the cytosolic side of the plasma membrane, subsequently being translocated on the mitochondrial side. These conclusions were drawn from the experiments that detect hydrogen peroxide and superoxide radicals extracellular formed using molecules that cannot cross the neutrophil membrane and therefore can only detect extracellular ROS. Luminol (5-amino-2,3-dihydro-1,4-phtalazinedione) detects the intra and extracellular ROS and lucigenin detects only extracellular superoxide [46].

It can be said that the biosynthesis of ROS by respiratory burst along with the release of proteolytic enzymes are important physiological defense mechanisms of phagocytic cells against pathogens, but in the absence of the antioxidant enzymes, the excess of free radicals may cause damage to the adjacent tissues.

The ability of peripheral blood mononuclear cells to produce free radicals versus their neutralizing capacity was determined by measuring the NADPH oxidase activity. Decrease in NADPH oxidase function impair the phagocytic immune cells to produce free radicals with microbicidal properties decreasing their ability to destroy pathogens end explaining the high rate of infections in diabetic patients [47].

Physiologically, the reactive process during phagocytosis fades slowly. But if the activity of antioxidant enzymes (modified by glycation) is reduced, respiratory burst persists and reactive oxygen species produced in excess causes oxidative damage in adjacent tissues. Phagocytosis is associated with an increase in the consumption of oxygen and the elimination of superoxide anion and hydrogen peroxide. Superoxide anions have a weak microbicidal action while hydroxyls possess a strong bactericidal effect, but affecting simultaneously the surrounding tissue. All these molecules lead to degranulation and activation of various proteases (elastase, collagenase and others) which will act both in terms of destruction of the aggressor, but will injure also the healthy surrounding tissue [48,49,50].

During this study we have performed an evaluation of adipose tissue secretory function parameters, which were further correlated with the anthropometric parameters as well as with those specific for beta pancreatic cells activity. The statistical analysis revealed that the obesity status and degree, total body fat mass and visceral fat level were correlated with an impaired pancreatic cell secretory activity which ultimately leads to insulin resistance. 

The BMI was positively correlated with the visceral fat level (*p* < 0.001, r = +0.61), the percentage of body fat mass (*p* < 0.001, r = +0.67), the C-peptide (*p* < 0.001, r = +0.39) and leptin (*p* < 0.001, r = +0.58).

A significant positive correlation was also found between leptin and body fat percentage (*p* < 0.001, r = 0.7), waist circumference (*p* 0.001, r = +0.33) and a negative correlation between adiponectin and visceral fat level (*p* < 0.001, r = −0.35). Also, a negative correlation was found between adiponectin and triglycerides/HDLc ratio (*p* < 0.001, r = −0.27), supporting the hypothesis that the type (particularly abdominal) and the degree of obesity is accompanied by increasing leptin and decreased adiponectin levels, both adipokines being secreted by the adipose tissue.

The percentage of total body fat was positively correlated with TOS (*p* < 0.001, r = +0.32) indicating that obesity is associated with increased oxidative processes and disrupted oxidant/antioxidant balance with a production of reactive species and a decrease in body's ability to neutralize them, all on the background of an pro-inflammatory state. Other studies have also revealed a correlation between body fat mass and intensity of total oxidative status and also between insulin resistance indicators, obesity markers and atherogenic index [51,52].Positive correlations of HOMA-IR index with both waist circumference (*p* < 0.001, r = +0.29), the proinsulin/adiponectin ratio (*p* < 0.001, r = +0.45) and triglyceride /HDLc ratio (*p* < 0.001, r = +0.32) are obvious arguments supporting the significant relationship between insulin resistance, pancreatic beta cells dysfunction and hypertrophic and “aggressive” fat cells.

The oxidative stress markers were correlated both positively and negatively with the specific parameters for evaluation of pancreatic beta cells. We found a positive correlation between TOS and HbA1c (*p* < 0.001, r = +0.32) on the one hand and glucose on the other hand (*p* < 0.001, r = +0.32). Negative correlations were found between GPx and glucose (*p* < 0.001, r = −0.31), GPx and HbA1c (*p* < 0.001, r = −0.34), and between GSH and fructosamine (*p* < 0.001, r = −0.27).

These correlations are in accordance with the scientific literature supporting the hypothesis that overproduction of reactive species (primarily ROS) during chronic hyperglycemia results in lower activity of glutathione and of key enzymes with antioxidant properties, like SOD and GPx [53,54,55]. As expected, a negative correlation of TAS with respiratory burst (*p* < 0.05, r = −0.24) proves that the antioxidant defense decreases with amplified production of free radicals through the respiratory burst. 

## 4. Materials and Methods 

A cohort of 115 patients, 55 females and 60 males (mean age 58.33 ± 9.87 years) newly diagnosed (within 6 months) with T2DM was selected from outpatients treated at the National Institute of Diabetes, Nutrition and Metabolic Disease (NIDNMB) “Prof. N.C. Paulescu”. The study was approved by the Ethics Committee of NIDNMB “Prof. N.C. Paulescu” and all participants signed an informed consent form before they were included in the study.

Diabetes was diagnosed according to the WHO and American Diabetes Association (ADA) criteria: serum fasting glucose ≥ 7.0 mmol/L (126 mg/dL), or 2 h serum glucose obtained after an oral glucose tolerance test (OGTT) ≥ 11.1 mmol/L (200 mg/dL), or a glycated hemoglobin (HbA1c) ≥ 48 mmol/mol (6.5%) or a random plasma glucose ≥ 11.1 mmol/L (200 mg/dL) in patients accompanied by classical symptoms such as polyuria, polyphagia, polydipsia as well as a weight loss [56].

The control group comprised 32 healthy subjects—17 females and 15 males (mean age 48.41 ± 15.10 years)—without any suspicion of diabetes or glucose intolerance (normal glycaemia as well as HbA1c < 44 mmol/mol).

The exclusion criteria were: renal diseases, urinary infections, hemodialysis treatment, acute ischemic cardiovascular disease, cardiac and cerebral stroke, epilepsy or other severe diseases (e.g., hepatic failure, cancer or gangrene), seric creatinine > 1.36 mg/dL (120 μmol/L) proliferative retinopathy or severe maculopathy and also, use of vitamins, minerals, or other supplements in the previous month, excessive alcohol consumption (ethanol > 20 g/day) and pregnant women.

After overnight fasting, blood samples were collected into vacuum tubes with no anti-coagulant (for routine blood tests and ELISA measurements) or with EDTA for isolation of peripheral blood mononuclear cells (PBMC) and for the assessment of fructosamine, glutathione (GSH), total antioxidant status (TAS) and total oxidant status (TOS). A volume of 10mL from each EDTA — containing sample was immediately used for isolation of PBMC for Respiratory Burst (RB) and glutathione, while serum in the remaining sample (10 mL) was collected after centrifugation and stored at −80 °C until required.

HbA1c assay that determines the level of hemoglobin glycation was determined by High Performance Liquid Chromatography (HPLC), a certified National Glycohemoglobin Standardization Program (NGSP) method and standardized by the Diabetes Control and Complications Trial (DCCT) on an automatic D-10 Hemoglobin Analyzer (Bio-Rad Laboratories, Inc., Clinical Diagnostics Division, Dubai, United Arab Emirates).Anthropometric measurements included: weight, height, BMI (kg/m^2^), fat mass (%), visceral fat level (%) and lean body mass (kg) assessed using a bioelectrical impedance analyzer (Body Composition Analyzer BC 418 MA, Tanita Corporation, Tokyo, Japan).

The systolic as well as the diastolic blood pressure were measured for all volunteers included in the study. Waist circumference (WC) which is a commonly used indicator to assess abdominal fat mass (subcutaneous and intra-abdominal fat) was measured using a centimeter.

Routine blood tests included the circulating levels of glycaemia *a jeun*, total cholesterol, high density lipoprotein -cholesterol (HDLc), serum triglycerides (TG), urea, creatinine, uric acid, albumin, total proteins, gamma glutamyl transferase (GGT), alanine aminotransferase (ALT) and aspartate aminotransferase (AST) were measured using current biochemical methods on an Eos Bravo Forte Analyzer (Hospitex Diagnostics, Florence, Italy). Serum concentrations of insulin, proinsulin, C-peptide, leptin and adiponectin were determined by ELISA on a Multiskan EX automatic 96 plate reader (model 355 from Thermo Electron Corporation, Thermo Fisher Scientific, Waltham, MA, USA) using commercially available kits (EIA-2935, EIA-1560, EIA-1293, EIA-2395 and respectively EIA-4177; from DRG Instruments GmbH, DRG International, Inc., Marburg, Germany) following the manufacturer’s guidelines. The coefficient of variation (CV) was 2.2%, 4.86%, 6.12%, 6.43% and 5.66% respectively. 

In this study we also determined the following parameters: total cholesterol/HDLc ratio, triglycerides/HDLc ratio also known as atherogenic index (A.I.), low density lipoprotein−cholesterol (LDLc) was calculated according to the Friedewald equation [57], Proinsulin/Insulin ratio, Proinsulin/Adiponectin ratio, Homeostasis Model for Insulin Resistance (HOMA-IR) was recorded as [glycaemia (mmol/L) × insulinemia (μU/mL)]: 22.5 [58] and Homeostasis Model for Beta cell function (HOMA-BETA) was recorded as HOMA-Beta = 20 × (insulin)/((glucose) − 3.5)%.

Fructosamine as an indicator of the degree of glycation of albumin was determined using the Spinreact (Girona, Spain) kit (CV = 1.79%) which is a colorimetric assay based on the ability of ketoamines to reduce nitrotetrazoliumblue (NBT) to formazan, measured photometrically at 546 nm in an alkaline solution. 

Plasma total oxidant status (TOS) and total antioxidant status (TAS) were determined using the kits PerOx (TOS/TOC) no. KC5100 (CV = 2.94%) and ImAnOx (TAS/TAC) no. KC5200 (CV = 3.99%) respectively, both photometric tests from Immundiagnostik AG (Bensheim, Germany).

Among the oxidative stress parameters the levels of glutathione (GSH) and the activity of the antioxidant enzymes superoxide dismutase (SOD) and glutathione peroxidase (GPx) were determined by spectrophotometric methods. Glutathione was determined on fresh whole blood samples collected in EDTA vacutainers.

The absorbances of the samples were read at a wavelength of 405 nm on the Multiskan EX automatic plate reader.

To determine the activity of the enzyme superoxide dismutase (SOD) the SOD 19160 WST Assay kit (Sigma Aldrich Chemie GmbH, Buchs, Switzerland) was used. To determine the activity of glutathione peroxidase (GPx) the Glutathione Peroxidase Cellular Activity Assay Kit (Sigma-Aldrich, St. Louis, MO, USA) was used according to the manufacturer's instructions. The respiratory burst (RB) was measured by chemiluminometric assay on peripheral blood mononuclear cells (PBMC) (mostly monocytes/macrophages) isolated from fresh whole peripheral blood collected on EDTA vacuum tubes.

PBMC were isolated by density centrifugation (1.077 g/mL) on a Ficoll-Paque™ Plus (from Sigma-Aldrich Co., St. Louis, MO, USA) at 630 *g* for 30 min. Cell viability determined by Trypan Blue exclusion was ≥ 90%. The ability to produce a RB was monitored by luminol and lucigenin enhanced chemiluminescence method [59]. In short, to PBMC (0.3 × 10^6^ cells) washed twice and resuspended in 1mL PBS, and dark-adapted luminol and lucigenin was added. After monitoring spontaneous chemiluminescence for 15 min, the RB was initiated by adding of 100 μL phorbol 12-myristate-13-acetate (PMA) (final concentration 5.4 μmol/L) and 100 μL opsonized zymosan (OZ) (final concentration 0.83 g/L) and the maximum chemiluminescence peak was recorded (Luminoskan Ascent^®^ 392, 96/384 microplate luminometer from Thermo Electron Corporation, Thermo Fisher Scientific, Waltham, MA, USA). Chemiluminescence production was expressed as the Relative Chemiluminescence Units over time (RLU × 60 min).

All reagents, including Dulbecco’s phosphate buffered saline (PBS), fetal bovine serum (FBS), Ficoll-Paque™ Plus, Trypan Blue, phorbol 12-myristate 13-acetate (PMA), luminol (5-amino-2,3-dihydro-1,4-phthalazinedione; LM), lucigenin (bis-*N*-methylacridinium nitrate), opsonized zymosan A from *Saccharomyces cerevisiae*, were purchased from Sigma Chemical Co. (St. Louis, MO, USA).

Statistical analysis: Differences between groups were analyzed with IBM SPSS Statistics Data Editor v19 software (IBM Inc., New York, NY, USA) using Student t-test on independent samples. Correlations between parameters were evaluated by the Pearson test performed on the whole group (both CG and ND-T2DM). A value for *p* < 0.001(**) and *p* < 0.05(*) was considered statistically significant. Testing the normality of the distribution of variables was performed using the Kolmogorov-Smirnov and Shapiro Wilk test. The same data from the same individuals are also being analyzed to produce a Normal Q-Q Plot. The variables without a normal distribution were log-transformed to normalize the distribution and subsequently analyzed. For uniformity, results were expressed as mean ± standard deviation (SD)/standard error of the mean (SEM) for all parameters.

## 5. Conclusions

Diabetes mellitus is accompanied by a pro-inflammatory milieu and an intensification of the oxidative stress, essentially due to a disruption of the pro-oxidant/antioxidant balance leading to the development of insulin resistance and dysfunction of pancreatic β cells and subsequently the occurrence of dysfunctions in the vascular endothelium. 

As a perpetual cause and effect cascade, oxidative stress is thus involved in diabetes etiopathogenesis, being maintained and enhanced by chronic hyperglycemia and glucose toxicity. The increase in the synthesis of glycation end products enhances the oxidative stress and the pro-inflammatory state, contributing to increased peripheral insulin resistance and pancreatic dysfunction. However, the interaction between diabetes, obesity associated with insulin resistance and oxidative stress mechanisms is incompletely elucidated due to the short life span of ROS causing oxidative damage, while the temporal sequence in which these processes intervene in the etiology, evolution and complications of T2DM is not yet unveiled. The results of our study revealed that the carbohydrates metabolism is positively correlated with the intensity of oxidative stress and negatively with the antioxidant components. The correlations identified within this study suggest the existence of a network of complex interactions between T2DM, obesity, insulin resistance and oxidative stress.

## Figures and Tables

**Table 1:** The values for anthropometric and clinical parameters for control group and T2DM patients.

Anthropometrical and Clinical Parameters	Control Group (CG)			ND-T2DM Patients (ND-T2DM)				*p* Value	
	Males N = 15	Females N = 17	Total N = 32	Males N = 60	Females N = 55	Total N = 115	CG vs. ND-T2DM	Males CG vs. Males T2DM	Females CG vs. Females T2DM
Age (years)	48.40 ± 12.70	48.41 ± 17.32	48.41 ± 15.10	59.23 ± 9.57	57.34 ± 10.18	58.33 ± 9.87	*p* < 0.001	*p* = 0.006	*p* = 0.057
Weight (kg)	81.32 ± 13.20	68.65 ± 20.26	74.33 ± 18.35	91.63 ± 13.37	83.63 ± 18.47	87.88 ± 16.39	*p* < 0.001	*p* < 0.05	*p* < 0.05
Waist circumference (WC) (cm)	97.75 ± 14.68	88.75 ± 16.64	91.75 ± 15.96	106.52 ± 7.48	102.37 ± 11.47	104.55 ± 9.77	*p* < 0.05	NS	NS
Body mass index (BMI) (kg/m^2^)	26.70 ± 3.23	25.96 ± 5.82	26.33 ± 4.78	30.50 ± 3.85	32.44 ± 6.06	31.43 ± 5.11	*p* < 0.001	*p* = 0.0015	*p* < 0.001
Fat mass (%)	23.53 ± 6.39	33.82 ± 9.01	29.41 ± 9.42	30.56 ± 5.26	41.96 ± 5.43	36.20 ± 7.82	*p* < 0.001	*p* = 0.003	*p* = 0.003
Visceral fat level (%)	9.75 ± 3.95	6.75 ± 4.26	8.04 ± 4.33	15.76 ± 4.19	11.02 ± 2.78	13.42 ± 4.27	*p* < 0.001	*p* < 0.001	*p* = 0.0013
SBP (mmHg)	120 ± 8.16	124.1 ± 10.83	122 ± 9.57	128 ± 8.65	129 ± 13.68	128 ± 11.35	*p* < 0.05	*p* = 0.005	NS
DBP (mmHg)	67.30 ± 10.12	72.50 ± 8.66	69.80 ± 9.63	74.64 ± 9.30	75.09 ± 9.08	74.87 ± 9.15	*p* < 0.05	NS	NS

Data are expressed as mean ± SD; SBP–systolic blood pressure; DBP–diastolic blood pressure; NS = not significant.

**Table 2:** The biochemical characteristics of T2DM patients versus healthy subjects.

Biochemical Parameters	Control Group (Healthy Subjects) (CG)	ND-T2DM Patients (ND-T2DM)	*p*Value Control vs. ND-T2DM
Glycemia a jeun (mg/dL)	96.20 ± 6.91	172.53 ± 74.29	*p* < 0.001
HbA1c (%)	5.62 ± 0.26	7.74 ± 2.03	*p* < 0.001
Fructosamine (µmol/L)	408.04 ± 82.39	572.32 ± 311.18	*p* < 0.001
Creatinine (mg/dL)	0.84 ± 0.17	0.85 ± 0.18	NS
Serum Urea (mg/dL)	29.78 ± 6.64	34.60 ± 9.53	*p* < 0.05
Serum total cholesterol (mg/dL)	211.20 ± 46.50	215.33 ± 52.72	NS
Serum HDLc (mg/dL)	51.55 ± 13.21	44.63 ± 12.31	*P* < 0.05
Triglycerides (mg/dL)	120.08 ± 45.24	180.36 ± 101.67	*p* < 0.001
Serum LDLc (mg/dL)	135.48 ± 47.53	136.09 ± 47.40	NS
Total Chol./HDLc	4.46 ± 1.64	5.12 ± 1.63	*p* < 0.05
Tg./HDLc (Atherogenic Index)	2.66 ± 1.78	4.67 ± 3.37	*p* < 0.001
AST (U/I)	22.99 ± 10.71	23.84 ± 12.95	NS
ALT (U/I)	25.20 ± 17.85	30.13 ± 15.35	NS
Uric Acid (mg/dL)	5.28 ± 1.99	5.99 ± 1.73	NS
GGT(U/I)	39.50 ± 9.15	46.27 ± 6.36	NS
Albumin (g/dL)	4.33 ± 0.27	4.31 ± 0.21	NS
Total Serum Proteins (g/dL)	7.00 ± 0.41	6.96 ± 0.46	NS

Data are expressed as mean ± SD; NS = not significant; AST—aspartate aminotransferase; ALT–alanine aminotransferase; GGT–gamma glutamyltransferase.

**Table 3:** The values of parameters relevant to the assessment of pancreatic cell function, adipose tissue and oxidative status.

Parameters	Control Group (Healthy Subjects) (CG)	ND-T2DM Patients (ND-T2DM)	*p*Value Control vs. ND-T2DM
Insulin (µUI/mL)	9.20 ± 4.96	13.74 ± 10.68	*p* < 0.001
HOMA-IR Index (%)	2.20 ± 1.23	5.74 ± 4.39	*p* < 0.001
HOMA-BETA Index (%)	102.87 ± 52.23	63.92 ± 6.97	*p* < 0.001
Proinsulin (pmol/L)	1.25 ± 0.49	6.69 ± 0.82	*p* < 0.001
Proinsulin/Insulin (P/I)	0.10 ± 0.005	0.53 ± 0.06	*p* < 0.001
Proinsulin/Adiponectin (P/A)	0.25 ± 0.07	1.81 ± 0.39	*p* < 0.001
C-Peptide (ng/mL)	3.34 ± 2.19	4.66 ± 2.92	*p* < 0.05
Leptin (ng/mL)	13.98 ± 3.19	17.50 ± 16.18	NS
Adiponectin (µg/mL)	13.08 ± 10.30	8.10 ± 7.57	*p* < 0.05
SuperoxideDismutase (SOD) (U/g Hb)	99.72 ± 22.95	95.72 ± 19.92	NS

Data are expressed as mean ± SD; NS = not significant; HOMA-IR: Homeostasis Model Assessment for Insulin Resistance; HOMA-BETA: Homeostasis Model for Beta cell function; PMA: phorbol 12-myristate 13-acetate; LM: 5-amino-2,3-dihydro-1,4-phthalazinedione; LG: bis-*N*-methylacridinium nitrate; OZ: opsonized zymosan.
